# Detection of *Mycobacterium tuberculosis* from the stool of HIV sero-positive individuals suspected of pulmonary tuberculosis

**DOI:** 10.1371/journal.pone.0177529

**Published:** 2017-05-19

**Authors:** Gizaw E. Abaye, Tamrat Abebe, Adane Worku, Debela Tolessa, Gobena Ameni, Adane Mihret

**Affiliations:** 1 Department of Medical Laboratory Science, College of Health Sciences, Arsi University, Asella, Ethiopia; 2 Department of Microbiology, Immunology and Parasitology, College of Health Sciences, Faculty of Medicine, Addis Ababa University, Addis Ababa, Ethiopia; 3 Aklilu Lemma Institute of Patho-biology, Addis Ababa University, Addis Ababa, Ethiopia; 4 Department of Biomedical Science, College of Health Sciences, Arsi University, Asella, Ethiopia; 5 Armauer Hansen Research Institute, Addis Ababa, Ethiopia; Hebrew University, ISRAEL

## Abstract

**Background:**

The impact of tuberculosis (TB) is exacerbated in Africa because of the human immunodeficiency virus (HIV) pandemic. Pulmonary tuberculosis (PTB) diagnosis is difficult in HIV-infected patients and negative sputum results are more common which leads to diagnostic delay and increases morbidity and mortality. Extra-pulmonary samples such as stool may be easier to obtain and our approach may therefore significantly improve PTB detection in people living with HIV.

**Objective:**

To detect *Mycobacterium tuberculosis* from the stool of HIV sero-positive individuals suspected of pulmonary TB.

**Method:**

A total of 117 HIV-infected individuals from three public health facilities in Addis Ababa, Ethiopia were enrolled consecutively in the study. Paired morning sputum and stool samples were simultaneously collected from anti-retroviral therapy (ART) naïve individuals living with HIV and suspected for PTB. The diagnostic accuracy of the smear microscopy, culture and region of difference (RD)9–based polymerase chain reaction (PCR) in stool was compared with the accuracy of sputum testing. Chi-square test and kappa value were used to compare different method used.

**Results:**

Sputum culture positivity for mycobacteria was confirmed in 33(28.2%) of the study subjects. Of 33 individuals positive for sputa culture, 10 individuals were observed to be stools culture positive. Of the 84 individuals negative for mycobacteria by sputum culture, three (3.6%) were stool culture positive and thus, the sensitivity and agreement between stool culture as compare to sputum culture were 30.3% and 0.33, respectively. Of 117 individuals, 11(9.4%) were sputum smear positive and of 11 sputum smear positive three were also stool smear positive. While of the 106 sputum smear negative individuals’, only one was stool smear positive resulting in 12.1% sensitivity and 0.18 agreements against sputum culture. On the other hand, the sensitivity of RD9-based PCR directly on stool was 69.7% by considering sputum culture as a reference standard. Moreover, RD9-based PCR directly on sputum detected 7(6.0%) individuals who were sputum culture negative for *M*. *tuberculosis*.

**Conclusion:**

*M*. *tuberculosis* was detected in stool of individuals living with HIV who were negative for sputum smear microscopy and culture. Hence, examination of stool samples alongside with sputum samples increases the detection of PTB in individuals living with HIV.

## Introduction

Tuberculosis (TB) remains a major global health problem and human immunodeficiency virus (HIV) infection has contributed to a significant increase in the worldwide incidence of TB. It causes ill-health among millions of people each year and ranks as the second leading cause of death from an infectious disease worldwide, after the HIV [1]. Worldwide, 13% of TB patients have HIV co-infection, and as many as 37% have HIV co-infection in parts of African Region, which accounted for 75% of TB cases among people living with HIV(PLHIV) worldwide. Ethiopia is among the countries most heavily affected by the HIV and TB. The World Health Organization has classified Ethiopia 10^th^ among the 22 high burden countries with TB and HIV infection in the world [1].

Tuberculosis is the commonest opportunistic infection and the number one cause of death in HIV patients in developing countries, and accounts for about 40% of all manifestations seen in HIV patients [2]. HIV/ AIDS fuels the TB epidemics in many ways, such as promoting progression to active tuberculosis by weakens their immune system, increasing the risk of reactivation of latent TB infection, as well as increasing chance of TB infection once exposed to tubercle bacilli [3]. The risk of developing active TB in HIV positive individuals is increased many fold despite antiretroviral chemotherapy [4, 5]. Tuberculosis may occur at any stage of HIV disease and frequently the first recognized presentation of underlying HIV infection [6, 7]. As compared to people without HIV, PLHIV have a 20-fold higher risk of developing TB [8] and the risk continues to increase as CD4 T cell counts progressively decline [6].

Unlike the straightforward diagnosis and typical presentation of pulmonary tuberculosis (PTB) in HIV sero-negative individuals, the diagnosis of PTB in HIV ⁄ AIDS is more difficult [9]. This might be associated with inability or difficulty for patients to produce a sputum sample, a problem that is particularly common in young children and HIV-positive patients [10, 11]. In these relatively immunodeficient patient groups, a diminished inflammatory response may inhibit sputum production. Induced sputum techniques [12], nasopharyngeal aspirates [13], fiber-optic bronchoscopy [14], or the string test [15] may all be used to retrieve pulmonary secretions from patients unable to provide a sputum sample but may cause logistical, cost, or biosafety challenges. These limitations in the diagnosis of tuberculosis necessitate the development of new tests to identify *M*. *tuberculosis* in samples that can be obtained more easily. Stool holds promise for PTB diagnosis in patients who are unable to produce sputum and potentially avoid more invasive procedures [16, 17, 18].

Though sputum smear has traditionally been used as the method for making an early diagnosis of PTB but smear-negative PTB is more common in HIV-infected patients and leads to diagnostic delay [19]. Sputum culture is a more sensitive method of diagnosing PTB in such cases, but can take up to 8 weeks before a result is available. The patient’s condition invariably deteriorates during this interval. Other factors contributing to diagnostic delay are that patients with HIV-associated PTB present more commonly with atypical or normal chest radiographs [20, 21]. This diagnostic delay also results in increased hospitalization and increased costs to the health system. It has also been proposed that delay in the initiation of TB treatment may accelerate HIV infection [22].

It is imperative that efforts be made to expedite the diagnosis of TB in HIV-infected people. Therefore, examination of stool might offer an alternative method for TB diagnosis when sputum is difficult to obtain from PLHIV. Tuberculosis bacteria are believed to be transported from the lungs to the Oropharynx and swallowed since peristalsis from the broncho-tracheal tree occurs regardless of coughing and is part of normal physiology [23, 24, 25]. Therefore, examination of stool specimens may facilitate PTB diagnosis in PLHIV who are unable to produce sputum.

## Materials and methods

### Study design and subjects

An institutional based cross-sectional study was conducted on HIV sero-positive individuals attending three health facilities in Addis Ababa. Study subjects were enrolled between January and July 2014 consecutively from the outpatient clinics of Zewuditu Memorial Hospital, Federal Police Hospital and Teklehayimanot Health Center. Individuals ≥18 years old and with suspected PTB were considered to be the target study subjects. Study participants were required to be ART naı¨ve. Patients taking anti-tuberculosis treatment, ionized prevention therapy and suspected to have gastrointestinal TB excluded from study participation.

The study was approved by the Institutional Ethical Review Board and Research Committee of Microbiology, Immunology and Parasitology, Addis Ababa University. All patients involved in this report were provided a written informed consent prior to data collection. Records of patients were manipulated only by researchers. All samples were de-identified of personal identifiers for data entry and data analysis. *M*. *tuberculosis* positive cases were contacted with nurses and doctors for further management.

Each patient was instructed to provide paired morning sputum and stool for smear microscopy, culture, and RD9-PCR. Samples were kept at 4°C and transported to the Aklilu Lemma Institute of Pathobiology (ALIPB) TB laboratory within 48h of collection and processed for concentrated Ziehl-Nielsen, culture, and PCR.

### Microbiological procedures

After complete thawing and mixing, sputum specimens was digested and decontaminated by the N-acetyl-L-cysteine-NaOH method and centrifuged for 15 min at 3000g [26]. The concentrated sediment was used for smear microscopy, culture and PCR. The composite bacteriological methods (culture and/or smear microscopy) were considered as a reference standard. Isolates from the positive cultures was preserved with freezing media while at the same time heat killed in water bath at 80°C for 1 hour. The frozen and heat killed isolates was stored at -20°C for further molecular identification as described below.

One gram of stool specimens was emulsified with sterile glass beads in 10 ml Tris buffer, 0.05 M, pH 7.2. The preparation was then shaken thoroughly in order to mix the sample with the buffer solution and the suspension was filtered into a 50 ml conical centrifuge tube. About 5 ml of the stool filtrate was mixed with 3 vols 1% chlorhexidine digluconate (Sigma) [27, 28], vortexed for 15 min at room temperature, washed in phosphate buffered saline (PBS) and centrifuged for 20 min at 3000 *g* at room temperature. The pellet was suspended in 1 ml PBS for analysis.

A filtered 250μl stool or 250μl sputum sample was separately mixed with 500μl1×TE buffer (Tris—EDTA) and centrifuge for 20 minutes at 1200 g. The pellets were re-suspended in TE buffer, and 50μl of 10 mg/ml lysozyme was added, mixed well and incubated for 1h at 37°C. Seventy micro-liter sodium dedocylsulphate (Sigma, St. Louis, Mo.) with concentration of 10g/ml and 6μl of 10 mg/ml proteinase K (VWR international Ltd., poole, BH151TD, England) were then added, mixed, and incubated for 10 min at 65°C. Afterwards, 100 μl of 5 M NaCl was added and vortexed and following the addition of 80μl of pre-warmed cetyltrimethyl ammonium bromide (CTAB) /NaCl (Sigma, St. Louis, Mo.) in pure water, and the mixture was incubated at 65°C for 10 min. Approximately equal volume (700–800 μl) of readymadephenol:chloroform:isoamyl alcohol (VWR international Ltd., poole, BH151TD, England) in proportion of 25:24:1 was added, after vortexed for at least 10 seconds and centrifuging for 10 min at 12,000 rpm. The resultant upper phase was transferred to a clean tube with 0.6 volume of isopropanol and mixed gently. The tubes were then moved slowly upside down to precipitate the nucleic acid and incubated at -20°C overnight. Spun in a Microfuge for 15 min at 12,000 rpm, the precipitate was washed by 70% cold ethanol and the supernatant was removed. The pellet was permitted to air dry for 15 minutes and above. Finally, it was re-suspended in 1xTE buffer (Sigma, St. Louis, Mo.) solution (from 20μ to 50μ) based on the size of the pellet for RD 9 PCR amplification. Positive and negative controls were used in the whole procedures.

The genomes of the isolates were analyzed by PCR for the presence or the absence of regions of difference (RD 9) originally described as being deleted in the genomes of BCG isolates relative to the sequence of *M*. *tuberculosis* H37Rv[29, 30, 31]. A multiprimer PCR assay with three primers was used to detect RD 9 [29]. The internal control Known *M*. *tuberculosis* (MTB) was included in every PCR in order to check for the presence of PCR inhibitors while Qiagen water was used as negative control. The result was interpreted as *M*. *tuberculosis* (RD9 present) when a band of 306bp was observed comparing to commercially available ladder, divided by 100bp.

All data was entered into Epi Data version 3.1 and exported to SPSS software version 20 for analysis. Chi-square test (χ^*2*^) and kappa value were used to compare different method used. The sensitivity, specificity, negative and positive predictive were determined for microscopic examination of stool and bacteriological culture of stool by considering sputum culture as a gold standard.

## Results

A total of 117 eligible participants visiting out-patient clinics were enrolled. The participants’ age ranges from 19–61 years with a mean of 34.5 ± 8.89 and with male to female ratio of 0.63:1. Most of the participants were in the age category of 28–37 years 66(56.4%). Majority of the patients were married 78(66.7%) and reside in Addis Ababa 95(81.2%). Concerning the level of education and occupation of the respondents, most of them 67(57.3%) were in high school and governmental employee 56(47.9%). All the 117 subjects had cough complaint and majority of the study participants had night sweet 111(94.9%) and fever 99(84.6%). All patients diagnosed as pulmonary TB complaints cough, night sweat, and difficulty in breathing and none of them were positive for enlarged lymph node. The median body mass index was 20 (IQR = 18.3–22.0). Significant proportion 13(32.5%) of the PTB confirmed patients were at the stage II of the WHO definition. The median CD4 count was 195 (IQR = 93.5–285.5) cells ⁄μl.

### Detection rate of pulmonary tuberculosis

During the study periods, 117 paired stool and sputum samples from patients with suspicion of PTB were sent to the TB laboratory for comparative testing. Overall, 40(34.2%) and 28(23.9%) study participants were confirmed to be positive for *M*. *tuberculosis* from sputum and stool samples, respectively. Seventy seven (65.8%) of study individuals were negative by both sputum and stool samples (with mean age: 34.5 years; sex ratio male/female: 0.64:1) (Table 1). The rate of detection by smear, culture and PCR from both sputum and stool were 12(10.2%), 36(30.8%), and 40(34.2%), respectively. Out of 12(10.2%) individuals positive for smear microscopy, three (25.0%) patients were also positive for stool microscopy. Of the 36(30.8%) sputum and stool culture positive together, 23(63.9%) were positive by sputum culture, three (8.3%) were positive by stool culture while the rest 10(27.8%) were positive by both sputum and stool culture. Of 40(34.2%) patients positive by RD9-based PCR, 28(70.0%) patients excreted *M*. *tuberculosis* in both stool and sputum samples. However, 12(30.0%) of them only excreted *M*. *tuberculosis* in their sputum sample (Table 1).

**Table 1:** Rate of pulmonary tuberculosis by different tests carried out in study.

Sample(s)	Mycobacterial detection rate from specimens		
	Smear microscopy positive	L-J culture positive	PCR positive
Sputum (n = 117)	11(9.4%)	33(28.2%)	40(34.2%)
Stool (n = 117)	4(3.4%)	13(11.1%)	28(23.9%)
Both sputum and stool	12(10.2%)	36(30.8%)	40(34.2%)

L-J—Lewiston Jensen media; PCR—Polymerase chain reaction

### Bacteriological finding in the sputum

Of the 117 patients who provided sputa, pulmonary TB was confirmed bacteriologically in 33(28.2%) patients. From the 33 bacteriologically confirmed pulmonary TB patients, 11 (9.4%) patients were both smear and culture positive. The remaining 22 (18.8%) sputa were smear negative but culture positive. The detection of *M*. *tuberculosis* by culture was statistically significantly higher than that of the smear microscopic examination of sputum (χ^*2*^ = 27.1, p < 0.001). The RD9-based PCR detected 40(34.2%) in sputum specimen. Thus mycobacterial detection from sputum by RD9-based PCR was comparatively higher than that of the culture methods (χ^*2*^ = 84.4, p < 0.001) (Table 1). All sputum culture positive samples were positive by RD9-based PCR, and 22(55.0%) samples have discordant result between sputum smear and culture (Table 1).

### Bacteriological findings in the stool

From 117 patients stool, 13 (11.1%) were stools culture-positive. However, the culture isolation rate was significant (45.5%)(χ^*2*^ = 14.5, p<0.001) if the sputum samples from the same patient were also smear and culture positive as compared to those patients whose sputum samples were smear negative, culture negative, and RD9-based PCR positive (42.8%). From 4 of 117 patients confirmed to be positive by stool smear, it is also important to note that 4.5% of patients were found stool smear positive as compared with sputum smear negative, culture positive and RD9-based PCR positive. Therefore, the cumulative total PTB confirmed cases increased from 11(9.4%) to 12 (10.2%) and 33(28.2%) to 36(30.8%) by using stool smear and culture, respectively. Overall 28 of 40 (70%) sputum confirmed PTB patients were found RD9-based PCR positive. Stool RD9-based PCR positivity could reach as high as 81.8% in sputum smear-positive cases. However, the application of RD9-based PCR for stool samples was had highest diagnostic yield in sputum smear and/or culture confirmed patients as compared with smear and /or culture unconfirmed patients (χ^*2*^ = 41.9, p < 0.001) (Table 2).

**Table 2:** Mycobacteria detection rate of various in vitro diagnostic methods applied to stool specimen from PTB patients.

Suspected PTB cases(n = 117) (and result of sputum sample)	Detection rate of mycobacteria in stool		
	Microscopy	L-J culture	PCR
Smear, culture and PCR positive(n = 11)	3(27.3%)	5(45.5%)	9(81.8%)
Smear negative, culture and PCR positive(n = 22)	1(4.5%)	5(22.7%)	14(63.6%)
Smear negative, culture negative and PCR positive(n = 7)	0	3(42.8%)	5(71.4%)

Ten of 33 (30.3%) sputum culture positive samples were positive by stool culture, and 3 of the remaining 84(3.4%) sample has concordant result between stool culture as compare with sputum culture with sensitivity (30.3%) and specificity (96.4%). Twenty three of 33 (69.7%) sputum cultures positive samples were positive by stool RD9-based PCR and 5 of 84 patients were positive by stool RD9-based PCR with sensitivity (69.7%) and specificity (94.0%). The measures of agreement between stool smear, culture and RD9-based PCR as compare to sputum culture were 0.18, 0.33, and 0.67, respectively (Table 3).

**Table 3:** Comparison of mycobacteria detection rate of various in vitro diagnostic methods applied to stool specimen from PTB patients against sputum culture.

Stool	Sputum culture			Sensitivity %	Specificity%	PPV	NPV	Kappa Value
		Positive	Negative					
**Smear**	Positive	4	0	12.1	100.0	100.0	74.3	0.18
	Negative	29	84					
**Culture**	Positive	10	3	30.3	96.4	76.9	77.9	0.33
	Negative	23	81					
**PCR**	Positive	23	5	69.7	94.0	82.1	88.7	0.67
	Negative	10	79					

## Discussion

In this study, the performance of readily available clinical sample i.e., stool was evaluated against sputum specimen by different diagnostic methods. A total of 117 paired sputum and stool specimens were analysed for the presence of *M*. *tuberculosis* in 117 patients. Pulmonary tuberculosis was diagnosed in 40(34.2%) patients, including 36(30.8%) culture-positive patients and 12(10.2%) patients with microscopic detection of acid-fast bacilli (AFB) identified as *M*. *tuberculosis* by RD-9 PCR. Acid fast bacilli were found in 11(9.4%) sputum and in 4(3.4%) stool specimens. Molecular identification of AFB in stools was critical because *Mycobacterium avium* complex [32, 33, 34], *Mycobacterium*. *marinum* [34], *Mycobacterium florentium* [35] and *Mycobacterium gordonae* [33] have been previously detected in patients’ stools. Also, MTC organisms can be detected in the stools of patients with digestive tuberculosis [36], a situation that was not addressed in this study. The microbiological diagnosis of PTB in HIV infected patients was inefficient by conventional method and specimen. Sputum scarcity in HIV-infected individuals (especially with low CD4 T counts) hampers the diagnosis of PTB by conventional smear [37]. Thus, lack of sputum and the paucibacillary nature of TB in HIV infection can result in misdiagnosis or in classifying patients as smear-negative PTB [38]. According to our finding, parallel examination of stool and sputum samples increases smear positive cases PTB in HIV patients. In one patient became fecal smear positive unlike sputum smear though the sensitivity of 12.1% was very low which is concurrent with other studies [39, 40].

The rate of PTB detection from sputum and stool culture were 33(28.2%) and 13(11.1%), respectively. Though, routine culturing of feces for PTB detection was ineffective like sputum, the diagnosis of three participants (2.6%) for whom stool culture positive unlike sputum culture for PTB indicated that, if available, stool cultures may increase the number positive TB cases of PLHIV. This value must be counterbalanced against the increased processing requirements and higher culture contamination rates associated with culturing stool as also indicated by Oramasionwu et al [39]. Our finding was slightly higher than study by Khe´chine et al [40], in which MTC successfully grew in 9.7% of fecal sample from 134 patients suspected to be suffering PTB using the conventional solid culturing methods. In contrast to our finding fecal culture were detected higher PTB positive cases [41, 42]. The higher detection observed by this group (compared with our results) may be as a result of a larger volume of stool and improved decontamination/ concentration techniques could improve the sensitivity of stool culture. However, in this study, we used chlorhexidine method as previous study [40]. The sensitivity of 30.3% is too low to suggest that stool specimen should replace sputum specimens for TB diagnosis, and is lower than the sensitivity reported in other studies [39, 40].

The RD9-based PCR test detected *M*. *tuberculosis* DNA in 40(34.2%) and 28 (24%) patients sputum and fecal specimens. It showed lower sensitivity and specificity (69.7% and 94.0%, respectively) as compared with sputum cultures. The obtained results were lower than other studies [17, 18, 40, 43, 44]. Inhibitors were likely to be the cause of most false-negative fecal PCR results in our study. The RD9-based PCR internal control indicated that 12(10.2%) fecal specimens were partially inhibited, a high value when compared with a reported 2.2% rate of inhibition using fecal specimens [38]. This could be due to the DNA extraction protocol we adopted in this study could be inefficient in removing PCR inhibitors from stools. As a result, these factors could have affected the detection of the *M*. *tuberculosis* contained within the samples.

## Conclusion

The limitation of this study is the inclusion of one sputum specimen. Nevertheless, from our results, one can conclude that laboratory investigation of stools demonstrated potential utility for the diagnosis of TB, although they did not perform better than sputum. Sputum should remain the diagnostic specimen of choice for PTB; however, stool culture particularly valuable in patients unable to produce sputum specimens. Performing stool and sputum smear microscopy for such patient also unforgettable. The PCR is a potential diagnostic tool that can be used in the diagnosis of PTB in people living with HIV. However, its ultimate use in developing countries especially Ethiopia is depends on evaluation of its cost effectiveness for routine diagnosis.
